# Inhibition of phosphatidylcholine-specific phospholipase C downregulates HER2 overexpression on plasma membrane of breast cancer cells

**DOI:** 10.1186/bcr2575

**Published:** 2010-05-12

**Authors:** Luisa Paris, Serena Cecchetti, Francesca Spadaro, Laura Abalsamo, Luana Lugini, Maria Elena Pisanu, Egidio Iorio, Pier Giorgio Natali, Carlo Ramoni, Franca Podo

**Affiliations:** 1 Department of Cell Biology and Neurosciences, Section of Molecular and Cellular Imaging, Istituto Superiore di Sanità, Viale Regina Elena 299, 00161 Rome, Italy; 2 Section of Immunology, Istituto Tumori Regina Elena, Via delle Messi D'Oro 156, 00158 Rome, Italy

## Abstract

**Introduction:**

Overexpression on plasma membrane of human epidermal growth factor receptor 2 (HER2) is reported in 25% to 30% of breast cancers. Heterodimer formation with cognate members of the epidermal growth factor receptor (EGFR) family, such as HER3 and EGFR, activates abnormal cell-signalling cascades responsible for tumorigenesis and further transcriptional *HER2 *gene upregulation. Targeting the molecular mechanisms controlling HER2 overexpression and recycling may effectively deactivate this feedback-amplification loop. We recently showed that inactivation of phosphatidylcholine-specific phospholipase C (PC-PLC) may exert a pivotal role in selectively modulating the expression on the membrane of specific receptors or proteins relevant to cell function. In the present study, we investigated the capability of PC-PLC inhibition to target the molecular mechanisms controlling HER2 overexpression on the membrane of breast cancer cells by altering the rates of its endocytosis and lysosomal degradation.

**Methods:**

Localization on the membrane and interaction of PC-PLC with HER2, EGFR, and HER3 were investigated on HER2-overexpressing and HER2-low breast cancer cell lines, by using confocal laser scanning microscopy, flow cytometry, cell-surface biotinylation, isolation of lipid rafts, and immunoprecipitation experiments. The effects of the PC-PLC inhibitor tricyclodecan-9-yl-potassium xanthate (D609) on HER2 expression on the membrane and on the levels of overall HER2, HER2-HER3, and HER2-EGFR contents were monitored in the HER2-overexpressing SKBr3 cells, after either transient or continuous receptor engagement with anti-HER2 monoclonal antibodies, including trastuzumab. Changes of HER2 expression and cell proliferation were examined in SKBr3, BT-474, and MDA-MB-453 cells continuously exposed to D609 alone or combined with trastuzumab.

**Results:**

PC-PLC selectively accumulates on the plasma membrane of HER2-overexpressing cells, where it colocalizes and associates with HER2 in raft domains. PC-PLC inhibition resulted in enhanced HER2 internalization and lysosomal degradation, inducing downmodulation of HER2 expression on the membrane. Moreover, PC-PLC inhibition resulted in strong retardation of HER2 reexpression on the membrane and a decrease in the overall cellular contents of HER2, HER2-HER3, and HER2-EGFR heterodimers. The PC-PLC inhibitor also induced antiproliferative effects, especially in trastuzumab-resistant cells.

**Conclusions:**

The results pointed to PC-PLC inhibition as a potential means to counteract the tumorigenic effects of HER2 amplification and complement the effectiveness of current HER2-targeting therapies.

## Introduction

Mutation and dysregulation of epidermal growth factor receptor (EGFR) family members are related to cancer onset and progression [1,2]. In particular, overexpression of the protooncogene encoding for human epidermal growth factor receptor 2 (HER2 or ErbB2 or C-neu) is implicated in a variety of tumors [3,4], with an estimated prevalence of 25% to 30% in patients with primary or metastatic breast cancer [5] and reported poor prognosis [6-8].

Although lacking intrinsic ligand-binding capability, HER2 acts as the preferred partner for the formation of mitogenically active heterodimers with the cognate EGFR family members epidermal growth factor 1 (HER1 or EGFR), EGFR receptor 3 (HER3), and receptor 4 (HER4) [4,9,10], HER2-HER3 being the prevalent and most potent of these complexes [1,8,11]. HER2-containing heterodimers undergo slow endocytosis and more-rapid recycling back to the cell surface [12-14]. These features translate to potent mitogenic signal cascades involving multiple signalling pathways [15].

HER2 is therefore a relevant target for HER2-overexpressing breast cancer therapy. Current targeted treatments are based on the use of trastuzumab, a humanized anti-HER2 monoclonal antibody [16-22] or antibodies against other EGFR family members [23,24] or inhibitors of selective tyrosine kinase receptor phosphorylation sites [25-28].

An additional, still scarcely explored anti-HER2 treatment may selectively target molecular mechanisms controlling HER2 overexpression on the plasma membrane, its lysosomal pathway-dependent degradation [29], and recycling back to membrane domains [30]. By inhibiting signal-transduction cascades triggered by HER2 heterodimer formation and affecting the downstream events responsible for altered cell proliferation, survival, and gene overexpression [31], this approach might complement or alternate with the present therapy protocols, especially in cases of severe side effects (for example, cardiotoxicity) or onset of specific resistance to currently used agents [28,32,33].

In previous studies on aberrant phosphatidylcholine (PC) metabolism in cancer cells [34-38], we reported that inactivation of a 66-kDa PC-specific phospholipase C (PC-PLC) enzyme, recruited to the plasma membrane of mitogen-stimulated [39], cytokine-activated [40] and tumor cells [41], downmodulates the expression on membrane of specific receptors or proteins relevant to cell function.

The present work reports the first evidence on PC-PLC accumulation and association with HER2 on the plasma membrane of HER2-overexpressing breast cancer cells and on the effects of PC-PLC inhibition on HER2 internalization, degradation, and recycling and on cell proliferation after transient or continuous cell exposure to anti-HER2 monoclonal Abs, including trastuzumab.

## Materials and methods

### Antibodies and reagents

Rabbit polyclonal antibodies (pAbs) raised against bacterial (*Bacillus cereus*) PC-PLC and selectively cross-reacting with mammalian PC-PLC [42] was obtained and characterized as reported [39,43,44].

Anti-HER2 monoclonal antibodies (mAbs) 300G9 and W6/100 were developed by Dr. P.G. Natali at the Istituto Tumori Regina Elena (Rome, Italy). Rabbit anti-EGFR, anti-HER2, anti-HER3, and anti-Rab5B pAbs were purchased from Santa Cruz Biotechnology (Santa Cruz, CA, USA). Anti-β-actin mAb was from Sigma-Aldrich (St. Louis, MO, USA). Trastuzumab (Herceptin) and protease-inhibitor cocktail were from Hoffman-La Roche (Basel, Switzerland). Anti-Lamp-2 mAb was supplied by BD Biosciences (San Jose, CA).

Alexa Fluor-488 and -594 F(ab)_2 _fragments of goat anti-rabbit IgG (H+L), Alexa Fluor-488 and -594 F(ab)_2 _fragments of goat anti-mouse IgG (H+L) were purchased from Molecular Probes Inc. (Eugene, OR, USA), and goat anti-human FITC-conjugated from Cappel Co. (USA) were used as secondary Abs.

Goat anti-mouse and goat anti-rabbit IgG horseradish peroxidase (HRP)-conjugated antibodies and streptavidin-HRP were supplied by BioRad Laboratories, Inc. (Hercules, CA, USA).

Triton X-100, NHS-biotin, propidium iodide, tricyclodecan-9-yl-potassium xanthate (D609), 5-bromo-2'-deoxyuridine (BrdU), 3-(4,5-dimethyl-thiazol-2-yl)-2,5-diphenyltetrazolium bromide (MTT), and all other chemicals and biochemicals were from Sigma-Aldrich, unless otherwise specified.

### Cells

Nontumorigenic, immortal human mammary epithelial cell lines (MCF-10A and MCF-12A) and HER2-overexpressing (SKBr3, MDA-MB-453, BT-4T4) or HER2-low (MCF7, MDA-MB-231, MDA-MB-435) human mammary carcinoma cell lines were from American Type Culture Collection (ATCC, Rockville, MD).

MCF-10A and MCF-12A cells were cultured in DMEM F12 medium, supplemented with 5% horse serum, hydrocortisone (0.5 μg/ml), insulin (10 μg/mL), hEGF (20 ng/mL), and gentamicin/amphotericin-B (Gibco Laboratories, Grand Island, NY, USA). MCF7, MDA-MB-231 and MDA-MB-435 cells were grown in DMEM (Gibco Laboratories) supplemented with 1,000 g/L glucose and 10% foetal bovine serum (FBS). MDA-MB-453 and SKBr3 cells were cultured in DMEM supplemented with 1,000 g/L glucose/10% FBS and 4500 g/L glucose/5% FBS (Gibco), respectively. BT-474 cells were cultured in RPMI supplemented with 10% FCS. Cells were typically analyzed at subconfluence, 72 hours after seeding.

### Proliferation assay

Cell-proliferation/viability assays were performed on cells exposed for 2 hours to MTT (5 μg/mL in PBS) at 37°C, in 5% CO_2 _[45,46]. Optical absorbance was measured (in six replicates) at 595 nm with a Model 680 Microplate Reader (BioRad Laboratories).

### Western blot analyses

Preparation of total cell lysates, determination of protein concentration and Western blot analyses were performed as previously described [41]. Blots were incubated with anti-HER2 (Santa Cruz Biotechnology) or anti-PC-PLC pAbs. Densitometric analysis of protein bands was performed as reported [41].

### *In vitro *PC-PLC activity assay

PC-PLC activity was determined in whole-cell lysates by using the Amplex Red PC-PLC-specific assay kit (Molecular Probes Inc.), modified as described [47].

### Separation of lipid rafts by sucrose gradient

Cell lysis, gradient fractionation (5% to 30% sucrose), and protein separation were carried out as previously described [40,41,48]. The distribution of HER2 and PC-PLC in the gradient fractions was assessed by separation in 7% SDS-PAGE followed by Western blotting.

### Cell surface biotinylation

SKBr3 cells were biotinylated on plasma membrane by adding 1.5 mg of NHS-biotin that links to hystidine and lysine proteins residues, and then lysed in ice-cold lysis buffer and immunoprecipitated with anti-PC-PLC polyclonal Abs. Detection of biotinylated proteins was performed by using streptavidin-HRP.

### Immunoprecipitation

Samples for immunoprecipitation were prepared as previously described [40]. In brief, total cell lysates (1 mg in 1 mL) were incubated with 10% protein G Sepharose (Amersham Biosciences, Uppsala, Sweden) and with anti-HER2 (Santa Cruz Biotechnology) or anti-PC-PLC specific Abs overnight at 4°C. After extensive washing, beads were removed by centrifugation at 14,000 rpm and then SDS sample buffer 4×, containing 2-mercaptoethanol (ICN Biomedicals Inc., Irvine, CA, USA) was added to elute proteins by heating the sample at 100°C for 5 minutes. Immunoprecipitates were resolved by 7% SDS-PAGE under reducing conditions and blotted with related Abs.

### Confocal Laser Scanning Microscopy (CLSM) and flow cytometry analyses

CLSM observations were performed on either unfixed or fixed and permeabilized cells, as already reported [41]. Flow-cytometry analyses were performed as previously described [39].

### Statistical analysis

Data were analyzed by using GraphPad software, version 3.03. Statistical significance of differences was determined with one-way ANOVA or with Student's *t *test, as specified. Differences were considered significant at *P *< 0.05.

## Results

### Accumulation of PC-PLC on the outer-membrane surface of breast HER2-overexpressing epithelial tumor cells

**Figure 1:**
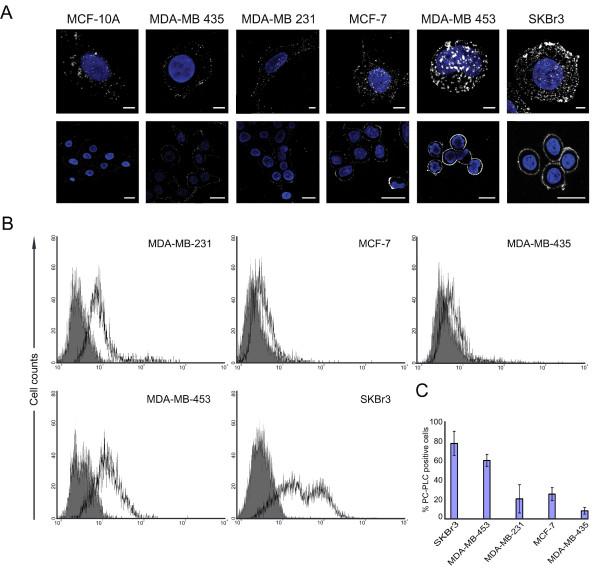
**PC-PLC expression on plasma membrane of breast cancer cells**. **(a) **CLSM analyses of six unfixed cell lines stained for PC-PLC detection (pseudo-color grey). Upper panels show examples of a three-dimensional reconstruction of a single cell (scale bars, 5 μm); the lower panels show single central sections of a group of cells (scale bars, 20 μm). Nuclei were stained with DAPI (blue). **(b, c) **FACS analyses of unfixed cells: representative cytofluorimetric histograms of PC-PLC relative fluorescence intensity of the indicated cell lines **(b) **and percentage (mean ± SD) of PC-PLC positive cells **(c)**. Statistical significance of differences between HER2-overexpressing (SKBr3 and MDA-MB-453) and HER2-non-overexpressing (MDA-MB-231, MCF-7, MDA-MB-435) carcinoma cell lines was *P *< 0.001. Five independent experiments were performed on each cell line.

CLSM and flow-cytometry analyses on unfixed cells showed barely detectable PC-PLC levels on the plasma membrane of the nontumoral immortalized breast epithelial cell lines MCF-10A (Figure 1a and 1b) and MCF-12A (data not shown). A massive, spotlike accumulation of PC-PLC was instead detected on the outer surface of the HER2-overexpressing breast carcinoma cell lines SKBr3 and MDA-MB-453. Similar fluorescence patterns were detected in BT-474 cells (Supplemental figure S1 in Additional file 1). In HER2-low cancer cells, PC-PLC-positive granules were smaller and less abundant (Figure 1a and 1b).

The percentage of PC-PLC-positive HER2-overexpressing cells (Figure 1c) was significantly higher (78% ± 13% in SKBr3; 61% ± 6% in MDA-MB-453; *P *< 0.001) than that of HER2-low tumor cells (MCF-7, 26% ± 7%; MDA-MB-231, 21% ± 15%; MDA-MB-435, 8% ± 3%) and nontumoral MCF-10A cells (<5% ± 2%).

These results show that PC-PLC selectively accumulates on the plasma membrane of HER2-overexpressing breast cancer cells.

### Colocalization and association of PC-PLC with HER2 on the plasma membrane of SKBr3 cells

**Figure 2:**
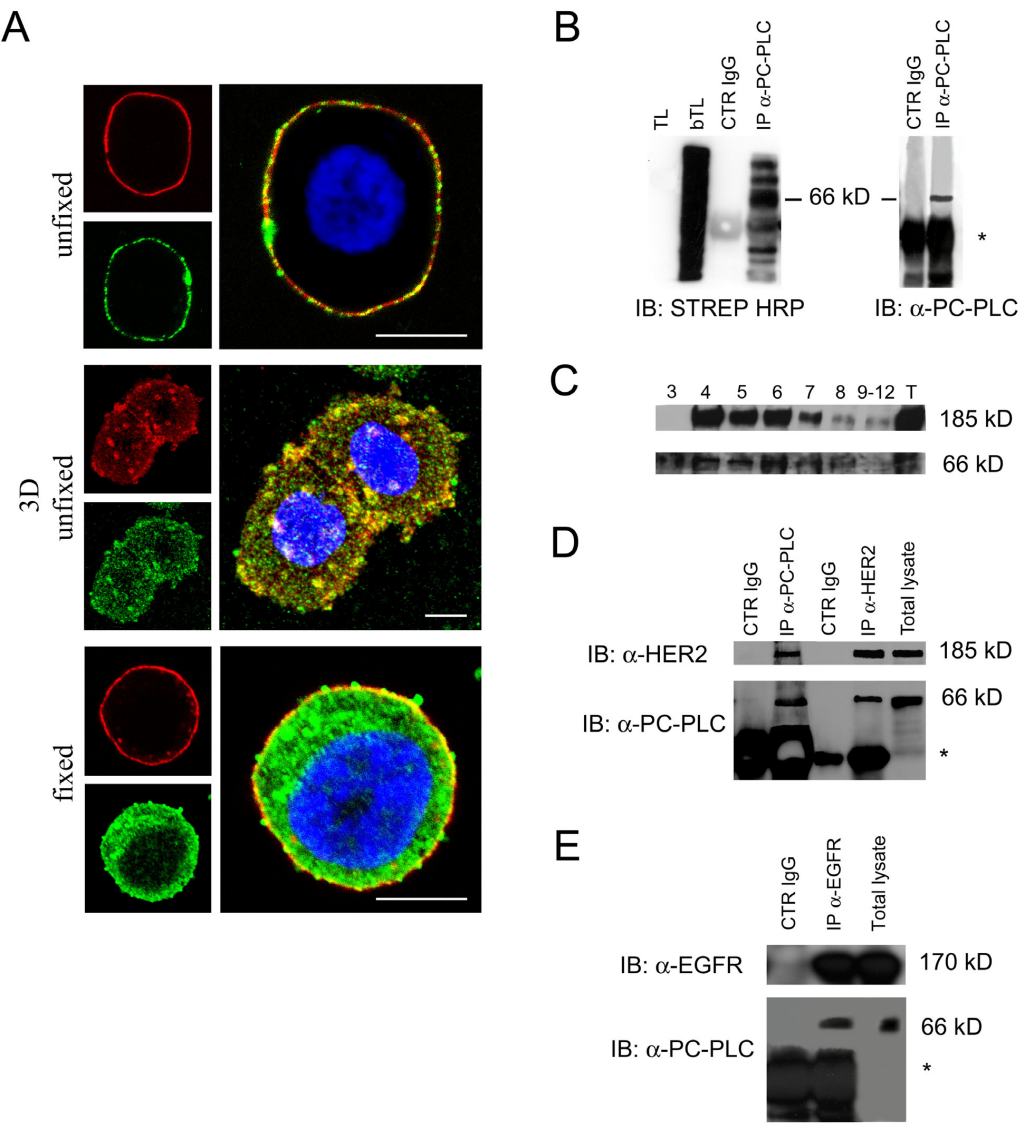
**Colocalization of PC-PLC and HER2 on plasma membrane of SKBr3 cells**. **(a) **CLSM detection of PC-PLC and HER2 in either unfixed (top and middle panels) or fixed and permeabilized SKBr3 cells (bottom panel) by using rabbit polyclonal α-PC-PLC (green) and α-HER2 W6/100 mAb (red). Colocalization areas are represented in yellow. The middle panel shows the tridimensional reconstruction of PC-PLC and HER2 expression on the plasma membrane. Scale bars, 8 μm. At least five independent series of experiments were performed for each condition. **(b) **PC-PLC immunoblotting analyses of immunoprecipitated biotinylated proteins isolated from SKBr3 cells, detected by streptavidin-HRP (left, IB:STREP HRP) or by α-PC-PLC (right, IB:α-PC-PLC). TL, total lysate; bTL, biotinylated total lysate; CTR IgG, control for α-PC-PLC IgG; IP α-PC-PLC, α-PC-PLC immunoprecipitates. *IgG heavy chains. **(c) **Sucrose gradient fractions isolated from SKBr3 cell lysates and analyzed by Western blotting for HER2 (185 kDa) and PC-PLC (66 kDa) detection. T, total cell lysate. **(d, e) **Western blot analyses of α-HER2 and α-PC-PLC **(d) **and α-EGFR and α-PC-PLC **(e) **immunoprecipitates (IP), blotted with the mutual Abs (α-PC-PLC, α-HER2, and α-EGFR, respectively), compared with the respective controls (CTR IgG α-PC-PLC, CTR IgG α-HER2, CTR IgG α-EGFR). Panels **b, c, d**, show representative results of three independent experiments. The immunoprecipitation in panel **e **was repeated twice.

CLSM analyses of unfixed SKBr3 cells showed extensive colocalization of PC-PLC and HER2 on the outer plasma membrane surface (Figure 2a, top and middle panels). Similar colocalization of the two proteins was observed on the plasma membrane of BT-474 cells (Supplemental figure S1 in Additional file 1).

Analyses of fixed SKBr3 cells showed that PC-PLC was also present in inner cell compartments including the nucleus (Figure 2a, bottom panel), whereas the HER2 receptor was, as expected, essentially confined to the plasma membrane.

Exposure of PC-PLC on the outer cell surface was confirmed by biotinylation on the membrane, followed by lysis and immunoprecipitation with anti-PC-PLC pAbs (Figure 2b, lane IP α-PC-PLC). Specific recognition by both streptavidin-HRP and anti-PC-PLC antibody of a biotinylated protein with a molecular mass of 66 kDa [39-41], identified the presence of a substantial fraction of PC-PLC on the outer membrane surface. Few additional bands detected at both lower and higher M_r _values were tentatively attributed to PC-PLC fragments and proteins associated with PC-PLC at the plasma membrane level.

Western blot analyses on sucrose-gradient fractions of total cell lysates showed that both HER2 (185 kDa) and PC-PLC were mostly present in detergent-insoluble membrane raft domains (Figure 2c, lanes 4 through 6), although low PC-PLC contents were also detected in the detergent-soluble fractions (Figure 2c, lanes 8 through 12).

Finally, combined co-immunoprecipation and Western blotting experiments showed that HER2 immunoprecipitates were associated with PC-PLC molecules (Figure 2d, lane IP α-HER2) and, conversely, a substantial amount of HER2 was present in PC-PLC immunoprecipitates (Figure 2d, lane IP α-PC-PLC).

Regarding other receptor-family members, EGFR (170 kDa, Figure 2e) but not HER3 (180 kDa, not shown) was also found to co-immunoprecipitate with PC-PLC.

These results provide evidence of the existence of a physical association between PC-PLC and HER2 (and EGFR) in HER2-overexpressing breast cancer cells.

### PC-PLC inhibition induces downmodulation of HER2 expression on the SKBr3 plasma membrane

**Figure 3:**
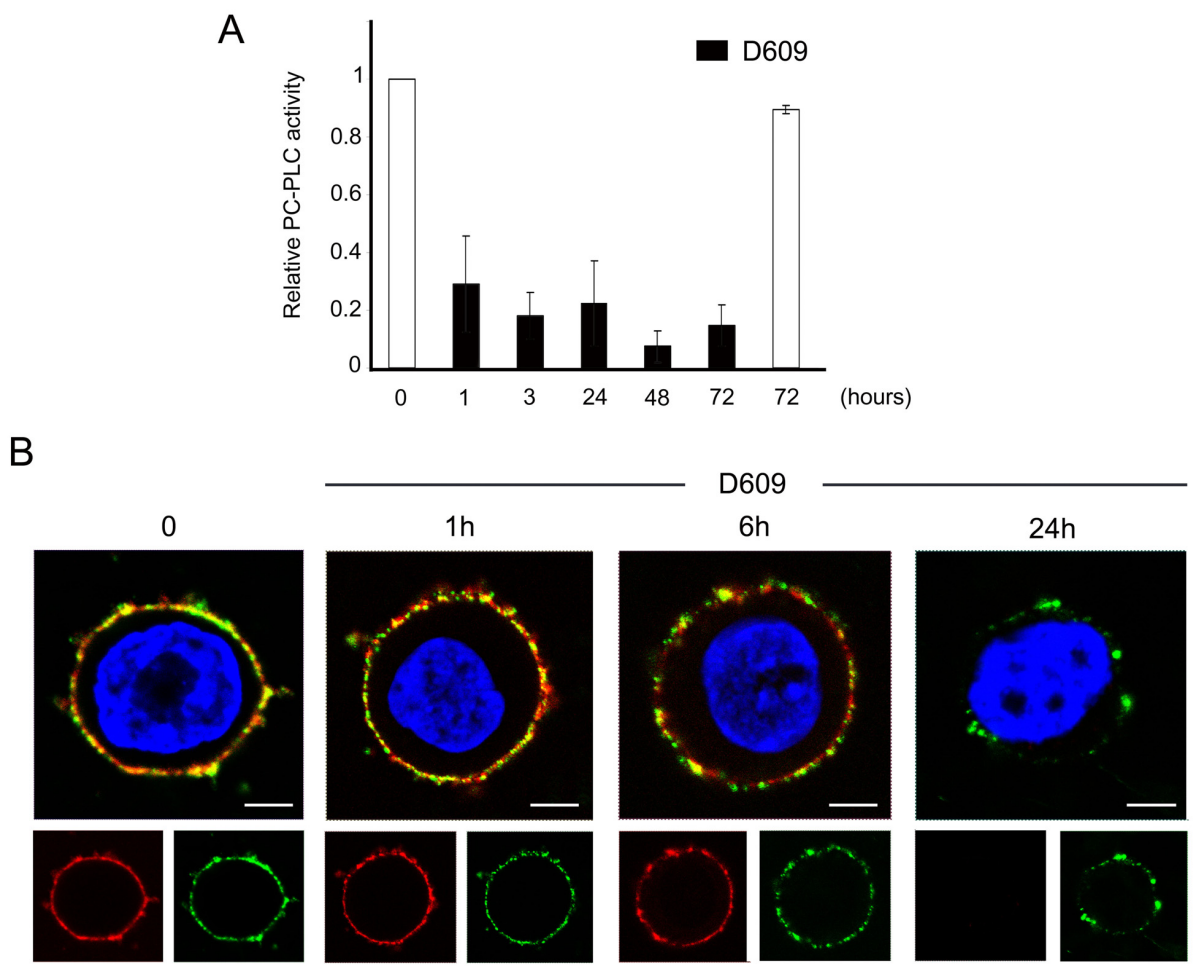
**D609-induced PC-PLC inhibition and HER2 downmodulation in SKBr3 cells**. **(a) **Amplex Red^® ^assay SKBr3 cells after exposure to the PC-PLC inhibitor D609. Relative PC-PLC activity was measured in the presence of D609 (50 μg/mL, black columns) and compared with that of untreated control cells (*t *= 0 taken as 1.0 or *t *= 72 hours, white columns). Histograms represent the mean ± SD (n = 4). **(b) **CLSM analyses of unfixed SKBr3 cells cultured in complete medium (*t *= 0) and in presence of D609 for the indicated time periods. After washing, cells were stained with α-PC-PLC (green) and α-HER2 (red). Nuclei were stained with DAPI (blue). Colocalization areas are represented in yellow. Scale bars, 5 μm. These experiments were independently repeated 3 times.

Possible effects of PC-PLC inactivation on HER2 expression on plasma membrane were investigated on SKBr3 cells cultured in the presence of the PC-PLC inhibitor D609. A 3.5-fold decrease in PC-PLC activity was achieved within 1 hour and was maintained up to 72 hours of cell incubation with D609 (Figure 3a, black columns). CLSM analyses showed a progressive downmodulation of both HER2 (red) and PC-PLC (green) on the membrane surface of cells exposed to D609, an effect already evident at 6 hours, and almost complete at 24 hours (Figure 3b). At this time, a small residual amount of PC-PLC was still detected on the cell surface.

### Inhibition of PC-PLC induces HER2 internalization and block of the receptor recycling

**Figure 4:**
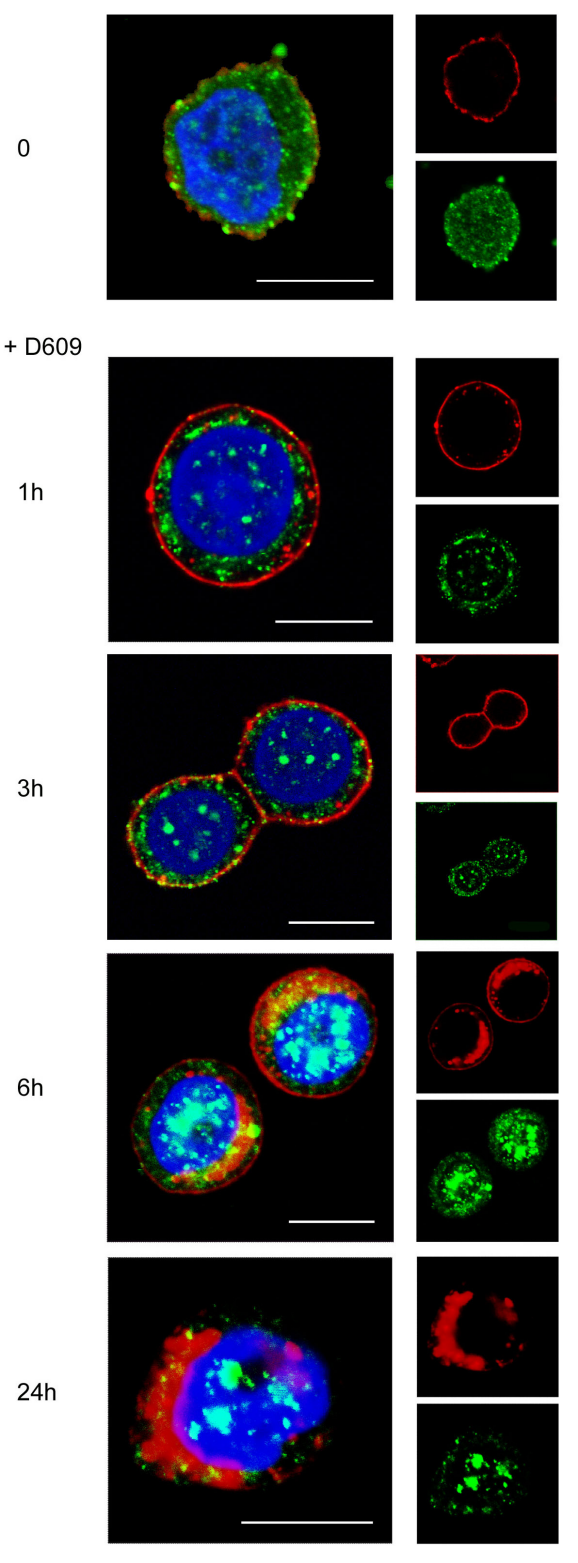
**Effect of PC-PLC inhibition on HER2 internalization in SKBr3 cells**. CLSM analyses of SKBr3 cells cultured in complete medium either in the absence (**t **= 0) or presence of D609 (50 μg/mL) for the indicated incubation times. After washing, cells were fixed and stained with α-PC-PLC (green) and α-HER2 Abs (red). Nuclei were stained with DAPI (blue). Colocalization areas were represented in yellow (merge between green and red) or cyan (merge between green and DAPI). Scale bars, 8 μm. Panels show representative examples of five independent series of experiments.

To investigate the effects of PC-PLC inhibition on HER2 expression on cell membrane, internalization, and recycling, CLSM analyses were performed on fixed and permeabilized SKBr3 cells after incubation in the presence of D609. A substantial amount of HER2 was internalized as small cytoplasmic vesicles within 1 hour of incubation (Figure 4), and at 6 hours, the receptor was mainly localized in large clusters close to the nucleus. Finally, at 24 hours, HER2 practically disappeared from the plasma membrane. The cytoplasmic PC-PLC distribution also underwent marked changes from a fine and diffuse distribution at t = 0 to large cytoplasmic granules at 6 hours, with progressive appearance of positive clumps inside the nucleus at 6 to 24 hours (Figure 4).

Similar HER2 downmodulation on the membrane was observed in BT-474 cells after exposure to D609 (Supplemental figure S2b in Additional file 2, column D609). Notably, these cells form clusters in which anti-HER2 Abs penetrate, allowing effective HER2 staining. However, small agents such as DAPI preferentially penetrate into peripheral cells (Supplemental figure S2a in Additional file 2). It is probably for this reason that the major effect of D609 was observed mostly on cells located at the periphery of BT-474 clusters.

The intracellular redistribution and trafficking of HER2 after D609-induced internalization was further investigated by CLSM on fixed and permeabilized SKBr3 cells stained with either the early endosomal marker Rab5B or the lysosomal-associated membrane protein-2 (Lamp-2), at different times of cell exposure to the PC-PLC inhibitor.

**Figure 5:**
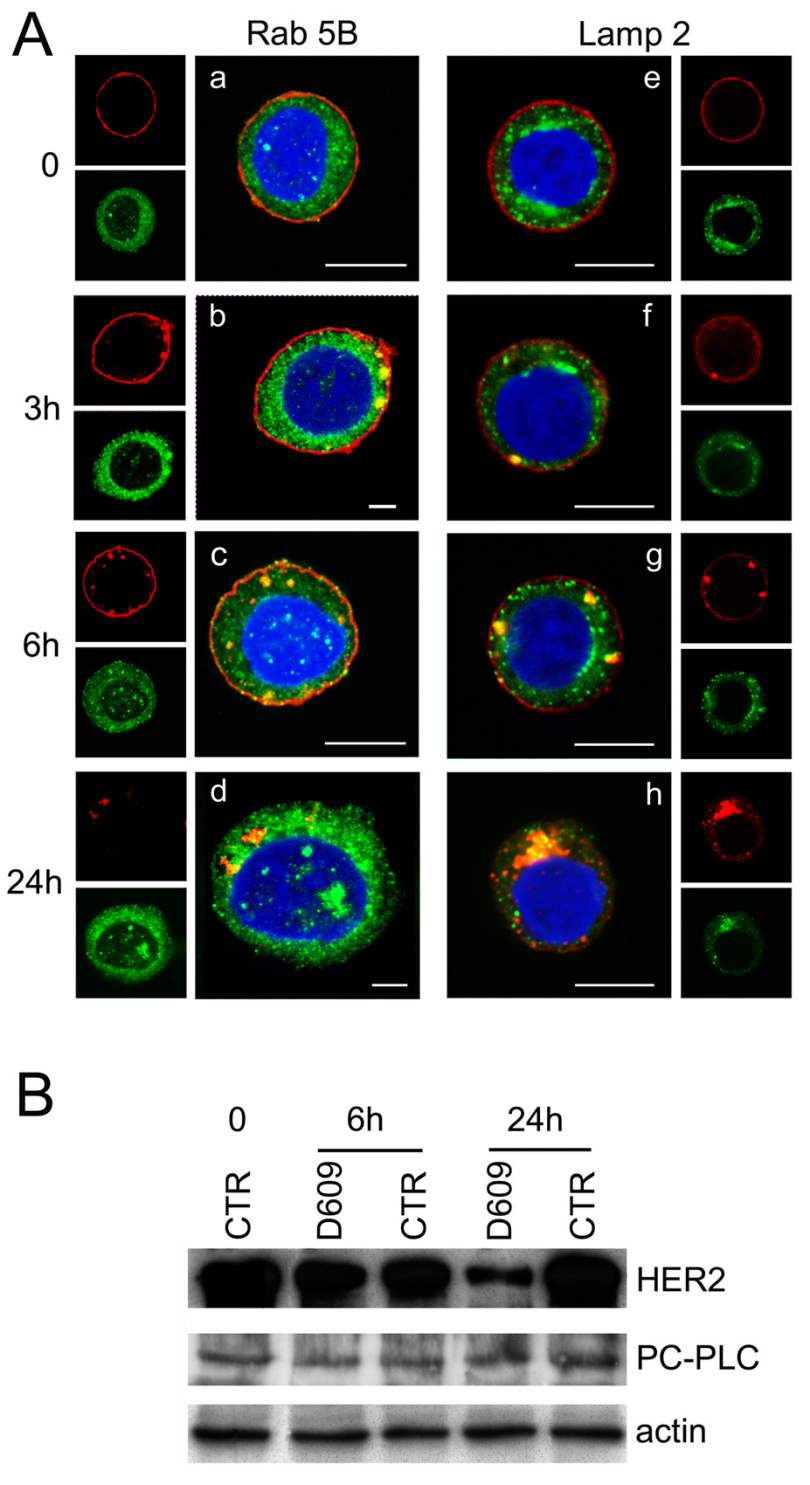
**Effects of D609 on trafficking and degradation of HER2 receptor molecules in SKBr3 cells**. **(a) **CLSM analyses of SKBr3 cells, incubated for the indicated time intervals with the PC-PLC inhibitor D609 (50 μg/mL) and then fixed and stained with the α-HER2 mAb W6/100 (red) and α-Rab5B Ab (green, panels **a **through **d**) or α-Lamp 2 Ab (green, panels **e **through **h**). Nuclei were stained with DAPI (blue). Scale bars, 8 μm. **(b) **Western blot analyses of total lysates of SKBr3 cells either untreated or incubated with D609 for 6 and 24 hours. The blots were incubated with α-HER2 or α-PC-PLC Abs. Actin was used as quantitative loading control. Experiments shown in panels **a **and **b **were independently repeated 3 and 4 times, respectively.

No evident colocalization of the receptor with either Rab5B- or Lamp-2-positive vesicles (Figure 5a, panels a and e, green) was observed at t = 0. In cells incubated with D609, most of internalized HER2 colocalized with early endosomes at 3 hours (panel b), only a small portion being associated with lysosomes (panel f). Both endosomal and lysosomal vesicles contained rather large HER2-positive clusters at 6 hours (panels c and g), whereas the receptor was mostly internalized into lysosomes at 24 hours (panels d and h).

Immunoblotting analyses showed a 70% decrease in the cellular HER2 content at 24 hours of cell incubation with D609 (Figure 5b), consistent with the enhanced receptor degradation in endosomes and lysosomes. No significant changes were detected in the overall cellular PC-PLC content.

These results showed that PC-PLC inhibition resulted in enhanced endosomal and lysosomal HER2 internalization, with downmodulation of this receptor on the plasma membrane.

### Effects of D609 on Ab-mediated HER2 internalization

**Figure 6:**
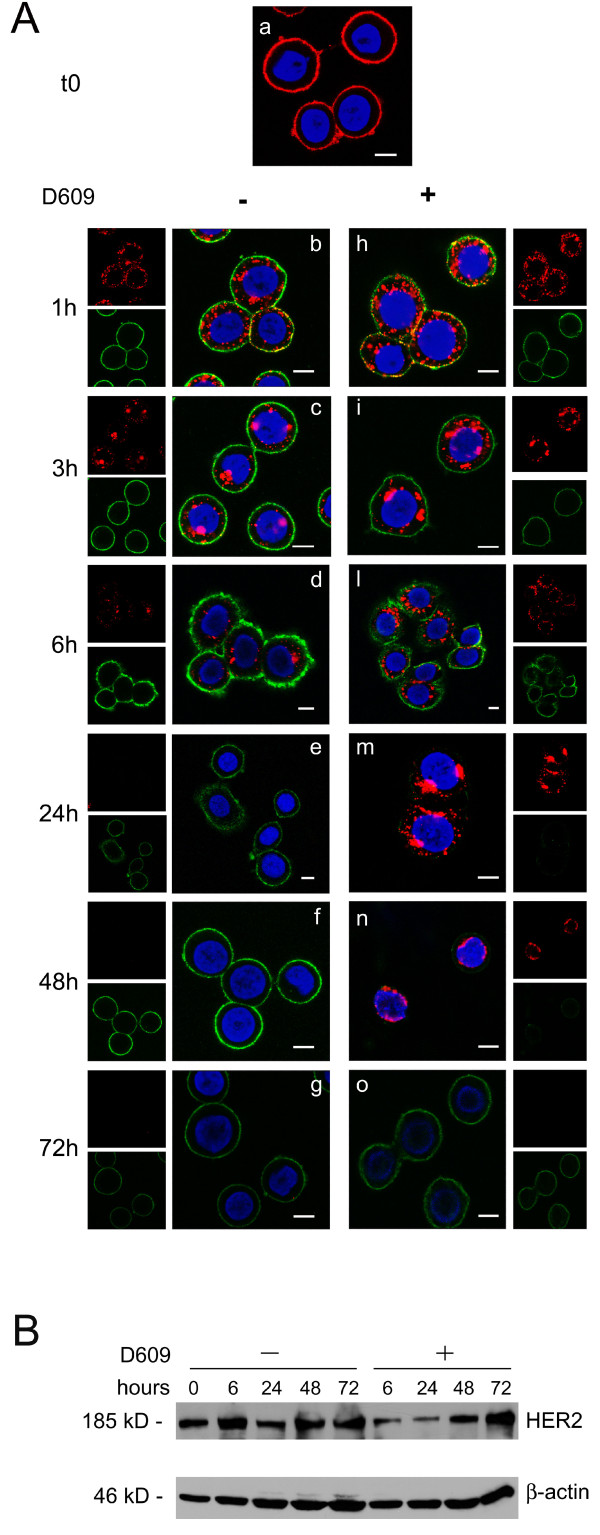
**D609-induced retardation of reexpression of HER2 on the plasma membrane of SKBr3 cells after short-term receptor engagement with an anti-HER2 antibody**. **(a) **CLSM examinations on unfixed cells transiently engaged (30 minutes at 4°C) with α-HER2 mAb 300G9, followed by goat α-mouse Alexa Fluor-594 (panel **a**, red), then cultured at 37°C for the indicated time periods in complete Ab-free medium, either in the absence (**b **through **g**) or presence of D609, 50 μg/mL (**h **through **o**). At the end of each time interval, cells were stained again on the plasma membrane with the α-HER2 W6/100 mAb, followed by goat α-mouse Alexa Fluor-488 (green). Nuclei were stained with DAPI (blue). Scale bars, 8 μm. These analyses were independently performed 3 times. **(b) **Western blot analyses of SKBr3 cells first engaged with α-HER2 300G9 mAb, followed by goat α-mouse antibody, and then cultured at 37°C either in the absence or presence of D609 for the indicated time periods. The blots were incubated with α-HER2 or with anti-β actin Abs. These experiments were repeated on seven sets of independent cell preparations.

HER2 engagement by specific Abs is known to induce receptor internalization from plasma membrane to inner cell compartments [29,49,50]. Experiments were therefore designed to explore whether PC-PLC inhibition could also interfere with the rates of Ab-mediated HER2 endocytosis, degradation, and reexpression on cell membrane. To this end, after short-time engagement with the anti-HER2 mAb 300G9 (30 minutes, on ice) followed by Alexa Fluor-594 Gα M Ab (red fluorescence), SKBr3 cells were incubated at 37°C in Ab-free medium in either the absence or the presence of D609 (Figure 6a). At selected time intervals, viable cells were stained again, at 4°C, on plasma membrane with a different anti-HER2 mAb (W6/100), followed by Alexa Fluor-488 as secondary Ab (green fluorescence), to monitor the time course of HER2 reexpression on the cell membrane (Figure 6a, green). Internalization of the "old" Ab-engaged receptor was therefore monitored in red, whereas reexpression on the membrane of "new" receptor molecules was monitored in green. Although the kinetics of HER2 internalization was likely altered by the presence of the secondary Ab, this experimental setting enabled us to investigate D609-induced changes in the repopulation of the plasma membrane with newly synthesized HER2 molecules.

Experiments on control cells (not exposed to D609) showed that a substantial amount of "old" HER2 receptor molecules was endocytosed within 1 hour from Ab engagement (Figure 6a, panel b, red), was mostly detected in cytoplasmic areas at 3 to 6 hours (panels c and d, red), and practically disappeared from the cell at 24 hours, likely because of lysosomal degradation (panel e, red). Reexpression on the membrane surface of "new" HER2 molecules was detected as early as 1 hour after Ab engagement (Figure 6a, panel b, green) and already returned to control values at 3 hours (panel c, green).

In the presence of D609, the rate of endocytosis of Ab-engaged HER2 molecules was quite similar to that of cells incubated in the absence of D609 but, interestingly, the cross-linked receptor underwent a much slower degradation; a portion of the originally engaged "old" HER2 molecules was still detectable in cytoplasmic areas at 48 hours (Figure 6a, panel n, red).

Furthermore, cell incubation with D609 induced a striking retardation in the reexpression of "new" HER2 molecules on the membrane surface (Figure 6a, panel l, green), because newly expressed receptor molecules were still below detection level at 24 to 48 hours (panels m and n, green), suggesting a long-lasting block of receptor recruitment to the cell surface. The effect of D609 on reexpression on the plasma membrane vanished at 72 hours of cell incubation with this inhibitor, when the "old" originally Ab-engaged receptor had been totally degraded (panel o, green and red staining, respectively).

Western blot analyses performed on total lysates of SKBr3 cells transiently engaged with the anti-HER2 Abs (Figure 6b) showed that the overall cellular HER2 content remained constant in the absence of D609, whereas it decreased to 52% ± 2% by 6 hours of cell incubation with D609, remained low at 24 hours, and gradually reverted to basal levels at 48 to 72 hours.

When HER2 was transiently engaged with the humanized antibody trastuzumab, the time course of receptor internalization (Supplemental figure S3 in Additional file 3, panels b through f, red) was similar to that observed in cells engaged with the 300G9 mAb (Figure 6, left panels) with only minor differences.

Overall, these results show that similar rates of HER2 internalization occurred under the adopted conditions in SKBr3 cells transiently cross-linked with a specific anti-HER2 Ab, irrespective of the presence of D609 in the medium. However, when incubation occurred in the presence of D609, the degradation of the originally Ab-engaged receptor was much slower, and reexpression on the membrane of new receptor molecules was strongly retarded (for at least 48 hours).

**Figure 7:**
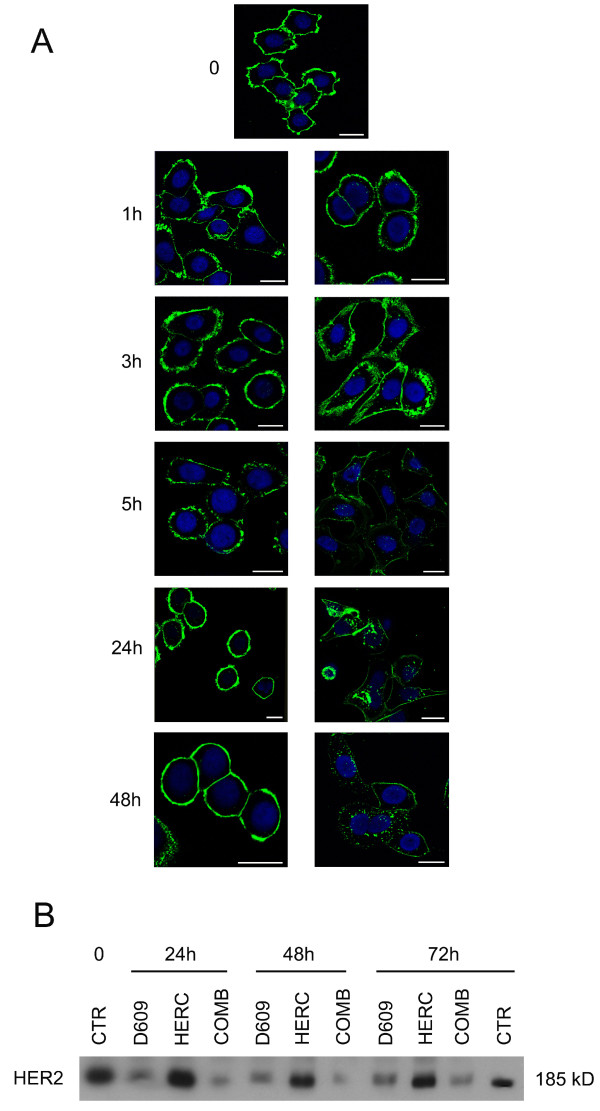
**Effect of D609 on HER2 expression in SKBr3 cells continuously exposed to trastuzumab**. **(a) **CLSM analyses of cells fixed after exposure for the indicated time intervals to trastuzumab, in the presence or absence of D609. Nuclei were stained with DAPI (blue). Scale bars, 20 μm. **(b) **Decrease in the overall HER2 content, detected by Western blotting, in total lysates of cells after incubation for different time intervals with D609 (50 μg/mL), trastuzumab (HERC, 10 μg/mL), or their combination (COMB). CTR, untreated control cells. Both CLSM and immunoblotting analyses were repeated 3 times.

When SKBr3 cells were continuously exposed to trastuzumab, CLSM analyses on cells fixed at the end of the incubation period failed to show any significant change in total HER2 expression on the membrane (Figure 7a, left panels), in agreement with a previous report [51]. However, in the presence of D609, a clearcut loss of HER2 was noted at the membrane level at 5 hours, which lasted for at least 48 hours (Figure 7, right panels). Immunoblotting experiments (Figure 7b) showed very substantial losses of overall HER2 content in subconfluent cells exposed to D609 (mean residual values versus controls of 67% ± 11% at 24 hours; 36% ± 8% at 48 hours; and 51% ± 18% at 72 hours) or to a combination of D609 and 10 μg/mL trastuzumab (54% ± 30% at 24 hours; 16% ± 1% at 48 hours, and 25% ± 6% at 72 hours), compared with trastuzumab alone (100% ± 4% at 24 hours; 81% ± 3% at 48 hours, and 79% ± 18% at 72 hours). Decreases in HER2 contents were also detected in MDA-MB-453 cells exposed to D609 (50% to 70% of control values at 24 to 72 hours) or to D609 combined with 50 μg/mL trastuzumab (30% to 60%) compared with trastuzumab alone (80% to 60%). These results showed substantial D609-induced HER2 decreases in these cells basically resistant to trastuzumab [52]. D609, either alone or in combination with 10 μg/mL trastuzumab, also induced a decrease in the HER2 content of trastuzumab-sensitive BT-474 cells at 72 hours (40% ± 10%), an effect comparable to that of trastuzumab alone.

### PC-PLC inhibition reduces membrane expression of EGFR and HER3 and formation of their heterodimers with HER2 in SKBr3 cells

To investigate whether PC-PLC inhibition could also induce alterations on membrane expression of other EGFR family members and formation of their heterodimers with HER2, combined CLSM, immunoblotting, and immunoprecipitation experiments were performed on SKBr3 cells incubated either in the presence or the absence of D609.

**Figure 8:**
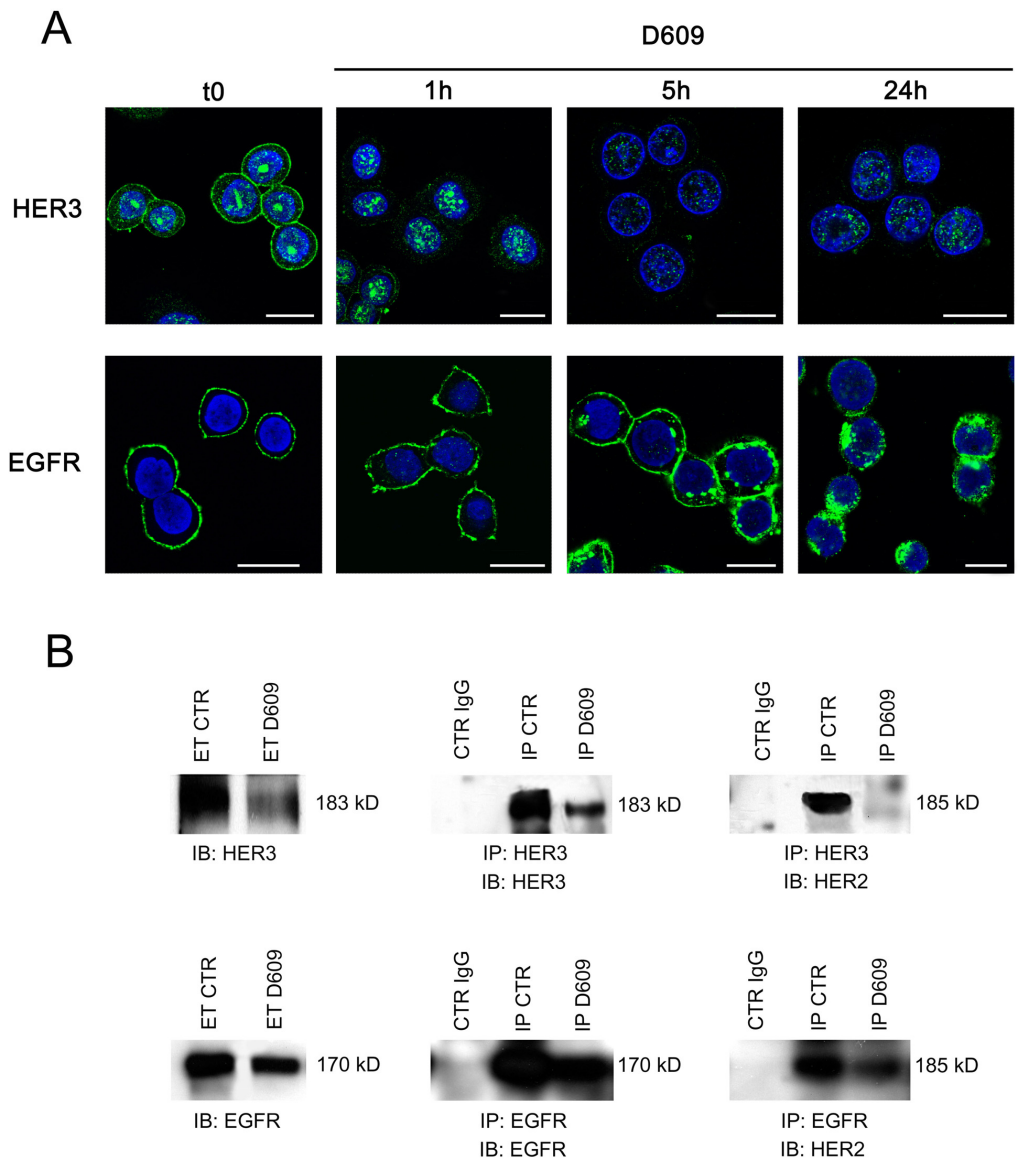
**Effects of PC-PLC inhibition on EGFR and HER3 internalization, overall receptors' contents and heterodimer formation with HER2**. **(a) **CLSM analyses of fixed SKBr3 cells after exposure to D609 for different time intervals and stained with either α-HER3 (upper panels) or α-EGFR antibodies (lower panels). Nuclei were stained with DAPI (blue). Scale bars, 20 μm. **(b) **Immunoblotting (IB) and immunoprecipitation (IP) experiments on SKBr3 cells, incubated with D609 for 24 hours, to detect overall HER3 and EGFR contents (left panels) and their levels of heterodimerization with HER2 (right). Central panels show the efficiency of the α-HER3 and α-EGFR antibodies in immunoprecipitating the respective target receptors. ET CTR, total extract of control cells; ET D609, total extract of D609-treated cells; CTR IgG, control IgG of α-HER3 or α-EGFR antibodies; IP CTR, immunoprecipitation from control cells; IP D609, immunoprecipitation from D609-treated cells. Representative examples of two independent experiments are shown.

CLSM analyses of untreated and fixed SKBr3 cells showed that, as expected, both HER3 and EGFR were expressed mainly on the plasma membrane, although the two receptors were differently distributed among inner cell compartments (Figure 8a). A substantial amount of HER3 (but not EGFR) was also detected in nuclear areas.

Cell exposure to D609 for 24 hours induced downmodulation from the plasma membrane of both HER3 and EGFR, although the two receptors followed different rates of internalization (Figure 8a). In particular, HER3 almost disappeared from the plasma membrane within 1 hour of cell exposure to D609, and only a small residual amount, confined mainly to the nucleus, was detected at 24 hours. At the latter time point, immunoblotting experiments on total cell lysates confirmed a strong decrease in the overall HER3 content (Figure 8b). Under these conditions, immunoprecipitation with anti-HER3 followed by detection with anti-HER2 Abs showed that HER2-HER3 heterodimers, clearly detected in control cells, practically disappeared in D609-treated cells (Figure 8b). This result was further confirmed by immunoprecipitation with anti-HER2 followed by detection with anti-HER3 Abs (data not shown).

CLSM analyses on D609-treated cells showed that the rate of EGFR endocytosis was very similar to that of HER2 internalization (compare lower panels of Figure 8a with Figure 4). Under these conditions, a clear decrease in the overall EGFR content was detected with Western blotting of total lysates of cells collected after 24 hours of exposure to D609. Co-immunoprecipitation experiments showed a simultaneous decrease in HER2-EGFR heterodimers (Figure 8b).

In conclusion, these results showed that 24 hours of cell exposure to the PC-PLC inhibitor induced a substantial decrease in both HER2-HER3 and HER2-EGFR heterodimers, a result that could derive not only from reduced HER2 expression (Figure 5), but also from the decreased contents of the respective partners.

### Effects of D609 on cell proliferation of HER2-overexpressing breast cancer cells

**Figure 9:**
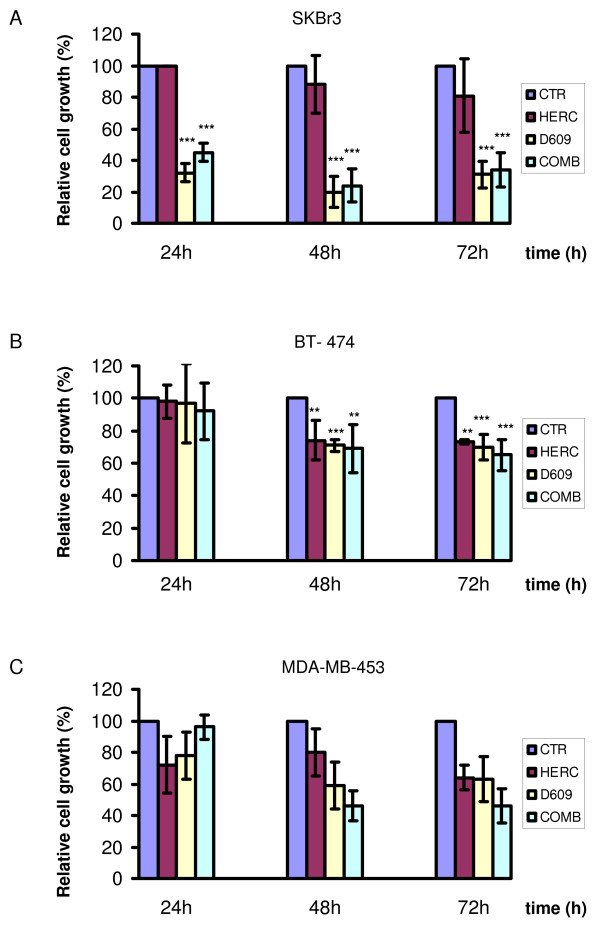
**Antiproliferative effect of PC-PLC inhibition on HER2-overexpressing cells**. MTT assays on **(a) **SKBr3 and **(b) **BT-474 cells both exposed to trastuzumab (HERC, 10 μg/mL), or **(c) **MDA-MB-453 exposed to trastuzumab (50 μg/mL), after incubation for the indicated times with D609, trastuzumab (HERC), or their combination (COMB). The percentage of cell proliferation was calculated by defining the absorption of untreated cells as 100%. Histograms represent the mean percentage of independent series of assays (± SD for SKBr3 and BT-474 (n = 3); or ± maximum deviation for MDA-MB-453 (n = 2)). Significance of differences: **P ≤ 0.02; ***P < 0.005.

MTT assays showed that trastuzumab failed to exert significant antiproliferative effects (*P *> 0.05) on SKBr3 cells continuously exposed for 24 to 72 hours to this agent at doses of either 10 μg/mL (Figure 9a) or 50 μg/mL (data not shown).

A strong reduction in the percentage of viable/proliferative cells was instead detected in SKBr3 cells exposed to D609 for 24 to 72 hours (down to 20% to 30% of the original levels in untreated control cells; *P *< 0.005). Combination of D609 with trastuzumab (10 μg/mL) had similar antiproliferative effects to those induced by D609 alone (Figure 9a). Different antiproliferative patterns were detected in the trastuzumab-sensitive BT-474 cells, in which this mAb reduced to 70% to 75% the percentage of viable/proliferative cells at 48 to 72 hours (*P *< 0.02). Very similar antiproliferative effects were induced by D609 or a combination of the two agents (Figure 9b), although it cannot be excluded that differential penetration of D609 in BT-474 clusters led to less-efficient PC-PLC inhibition.

Intermediate antiproliferative effects were exerted at 48 to 72 hours by D609 or D609 plus trastuzumab (50 μg/mL) in MDA-MB-453 cells (Figure 9c).

In conclusion, evident antiproliferative effects were associated with PC-PLC inhibition, especially in trastuzumab-resistant cells.

## Discussion

This study provided the first evidence that (a) PC-PLC, a phospholipase involved in mitogenic response, cell signalling, apoptosis, and survival [53-56], colocalizes with HER2 in membrane-raft domains of HER2-overexpressing breast cancer cells; and (b) PC-PLC inhibition induces in these cells enhanced HER2 internalization, followed by long-lasting HER2 accumulation in endosomal/lysosomal compartments and retarded HER2 reexpression on membrane. Moreover, PC-PLC was found to be physically associated with both HER2 and EGFR, but not with HER3 receptors, and PC-PLC inactivation resulted in striking decreases in the overall cellular contents of EGFR, HER2, HER3, and their heterodimers. Cell exposure to a PC-PLC inhibitor exerted an antiproliferative effect, especially on trastuzumab-resistant cells.

### Molecular interaction between HER2 and PC-PLC on the plasma membrane of SKBr3 cells

A previous study in our laboratory showed that inactivation of PC-PLC in human NK cells caused CD16 receptor downmodulation on membranes and reduced NK cytotoxicity [40]. Involvement of PC-PLC in the mitogenic response and growth-factor-dependent colocalization with β_1_-integrin was reported in ovarian cancer cells [41]. No information was available on subcellular localization and the role of PC-PLC in breast cancer cells, although preliminary evidence on activation of this enzyme has been reported [57]. The present study shows that PC-PLC accumulates on the cell surface of HER2-overexpressing cells, in which this phospholipase colocalizes with HER2 in membrane raft domains. Significantly lower PC-PLC levels were instead detected on the membrane of HER2-low breast cancer cells characterized by different malignancy phenotypes.

Colocalization of PC-PLC with HER2 on the plasma membrane of SKBr3 cells was due to physical association, because the two proteins were co-immunoprecipitated by the respective antibodies. Although identification of the chemical domains responsible for this molecular interaction needs elucidation of the structure of this still-unsequenced mammalian phospholipase, the association of HER2 with an active PC-PLC isoform is apparently an important requirement for maintaining this receptor anchored to functional membrane domains. Inhibition of PC-PLC activity was followed by fast downmodulation of the oncoprotein from the cell membrane, associated with endocytosis and targeting to degradative intracellular compartments.

The molecular mechanisms responsible for enhanced HER2 endocytosis and altered rate of degradation in HER2-overexpressing breast cancer cells exposed to the PC-PLC inhibitor remain to be elucidated. Previous studies by Kappler *et al*. [58] and Raja *et al*. [29] suggested a role for the endocytic pathway in HER2 downmodulation. In particular, a potent downregulation of the cell-surface HER2, associated with enhanced ubiquitinylation and lysosomal pathway-dependent degradation of this receptor and cell growth arrest was recently reported in HER2-overexpressing breast cancer cells treated with a combination of HSP90 inhibitor 17-AGG and trastuzumab [29]. The authors suggested that these agents may affect the interaction of HER2 with ubiquitin ligases, with subsequent changes in HER2 ubiquitinylation, by altering the arrangement of the HER2-associated HSP90 chaperone complex. These findings support the interest of exploring in our cell systems how deactivation of HER2-associated PC-PLC affects the differential recruitment of specific ubiquitin ligases to HER2 and cognate members of the EGFR family. We might hypothesize that deactivation of PC-PLC isoform(s) associated with HER2 likely modifies the efficiency of ubiquitinylation and lysosomal degradation through changes in steric hindrance effects or structural changes of HER2 complexes or both.

Our results point to the attractive possibility of controlling, by a simple switch of the activity status of PC-PLC, the levels of HER2 expression on the membrane and therefore reducing the formation of HER2 heterodimers with cognate members of the EGFR family, with expected effects on the control of tumorigenicity of HER2-overexpressing cells. By enhancing HER2 endocytosis and lysosomal degradation and retarding HER2 recycling to the membrane, PC-PLC inhibition resulted in HER2-EGFR and HER2-HER3 depletion. Similar effects on HER2 heterodimers have so far been achieved by more-complex laboratory procedures such as induction of intracellular expression of single-chain HER2-specific antibodies in cancer cells [59,60]. The D609-induced reduction in the levels of HER2-HER3 and HER2-EGFR heterodimers may originate from differential mechanisms, because co-immunoprecipitation experiments showed the physical association of PC-PLC with either HER2 or EGFR but not with HER3. Possibly linked to this differential behavior is the much faster downmodulation of HER3 from the cell membrane after PC-PLC inactivation. This result suggests the hypothesis that inhibition of PC-PLC activity may facilitate dissociation of HER2-HER3 heterodimers, with possible effects on diversification of HER2-driven intracellular cascades of downhill phosphorylation reactions [11]. Analyses should also be extended to HER4, temporarily excluded from this phase of the study.

### Role of PC-PLC activity in controlling HER2 recycling

Alterations of cell recycling of HER2 in breast cancer cells may also have profound effects on cell-signalling deregulation in HER2-overexpressing cells [12-14]. The reported downmodulation of HER2 from the plasma membrane of HER2-overexpressing cells exposed to the PC-PLC inhibitor suggests that PC-PLC has a role in controlling the effects of the overall HER2-amplification machinery. Our experiments on cells engaged by anti-HER2 Abs showed that fast receptor internalization and degradation was followed by rapid reexpression of new HER2 molecules on the membrane surface, consistent with the capability of these cancer cells to restore and maintain high expression levels of the amplified HER2 receptor. In contrast, addition of D609 to cells incubated in Ab-free medium after transient receptor-mAb cross-linking, not only induced further receptor endocytosis, and prolonged receptor degradation within lysosomal structures, but also slowed reexpression of newly synthesized HER2 molecules on the membrane. When cells were continuously exposed *in vitro *to trastuzumab (a condition similar to that of tumor cells after intravenous infusion of this agent to the patient), no substantial change in the expression of HER2 on the plasma membrane was observed. This effect was in good agreement with a previous report by Longva *et al*. [51] showing a substantial lack of endocytotic downregulation of HER2 in SKBr3 cells exposed to trastuzumab. When, however, cells were exposed to both trastuzumab and D609, a clear decrease in HER2 expression on the membrane lasted for at least 48 hours. Furthermore, a most striking effect induced, under these conditions, by the PC-PLC inhibitor was a strong and prolonged decrease in the overall cellular HER2 content, which lasted up to 72 hours.

### Reduced cell proliferation of SKBr3 cells exposed to the PC-PLC inhibitor

Among the multiple antitumor activities exerted by trastuzumab on HER2-overexpressing breast cancer cells, inhibition of cell proliferation is reported to occur primarily by modulation of the cyclin-dependent kinase inhibitor p27^Kp1^, via a complex network of signalling pathways, resulting in G_1 _arrest and growth inhibition [61].

This work shows a substantial lack of synergism between the antiproliferative effects exerted by trastuzumab and D609, these processes likely being regulated by different, independent molecular mechanisms. Moreover, the antiproliferative effect of D609 can hardly be regarded as specific for HER2-overexpressing cells, because previous studies in our laboratory showed that cell exposure to this xanthate is able to induce cell-cycle arrest in G_0_/G_1 _in different types of cells, such as HER2-non-overexpressing ovary cancer cells and mitogen-stimulated fibroblasts [39,40]. Further studies are needed to investigate whether and to what extent the antiproliferative effect of D609, similar to that of another PC-PLC inhibitor, SC-ααδ9 (4-(benzyl-(2- [(2,5-diphenyoxazole-4-carbonyl)amino]ethyl)carbamoyl)-2-decanolaminobutyric acid), is linked to the phosphorylation or inhibition or both of selective cyclin-dependent kinase activities [62].

Although a number of PC-PLC inhibitors have been identified or designed [63-65], D609 is widely accepted as selective competitive inhibitor of PC-specific phospholipase both in reaction mixtures and in cells [66]. This compound, first introduced as antiviral and antitumoral agent *in vitro *and *in vivo *[66-70], has been shown to enhance the action of other drugs in the regression of various human tumor transplants in athymic mice [68], without side effects.

Our reported evidence on multiple effects induced by D609 on HER2 downmodulation from the plasma membrane suggests the interest of further evaluating in appropriate model systems the added value of including a PC-PLC inhibitor as an adjunct to the current therapies targeted against HER2-overexpressing breast cancer cells.

## Conclusions

A C-type phospholipase specific for the major phospholipid of eukaryotic cells associates with HER2 and EGFR receptors in HER2-overexpressing breast cancer cells. Inhibition of this enzyme induces downregulation of HER2 expression on the plasma membrane of these cells, promotes HER2 endocytosis and lysosomal degradation, induces retardation in HER2 recycling, and is associated with reduction of overall HER2, HER2-HER3, and HER2-EGFR contents.

The use of a PC-PLC inhibitor as a modulator of HER2 overexpression and internalization in breast cancer cells may allow a better elucidation of the complex, still incompletely understood molecular mechanisms underlying cell tumorigenicity associated with *HER2/neu *gene amplification.

The results of this study point to PC-PLC inhibition as a possible means to counteract the tumorigenic effects of HER2 amplification and complement the effectiveness of current HER2-targeting therapies aimed at increasing disease-free and overall survival of patients affected by HER2-overexpressing breast cancer.

## Abbreviations

CLSM: confocal laser-scanning microscopy; Lamp-2: lysosomal-associated membrane protein-2; MTT: 3-(4,5-dimethylthiazol-2-yl)-2,5-diphenyltetrazolium bromide; PC: phosphatidylcholine; PC-PLC: phosphatidylcholine-specific phospholipase C.

## Competing interests

The authors declare that they have no competing interests.

## Authors' contributions

LP and SC carried out the confocal laser scanning microscopy and the molecular studies and drafted the manuscript. FS carried out the PC-PLC activity assays. LA performed the acquisition and analysis of the flow-cytometry data. LL participated in the molecular studies. MEP and EI carried out the MTT proliferation assays. PGN participated in the overall study design and critically revised the manuscript. CR participated in the design of the study and in the interpretation of data. FP conceived the study, participated in its design and coordination, and helped to draft the manuscript. All authors read and approved the final manuscript.

## Authors' information

LP presented this work as her thesis for the degree in Biological Sciences at the University of Rome "La Sapienza"; she is now a PhD student and research fellow at the Istituto Superiore di Sanità (ISS), Rome. LA and MEP are PhD students and research fellows at the ISS. SC, FS, and LL are PhD researchers at the ISS. EI is researcher at the ISS. PGN acted as Director of the Immunology Section at the Istituto Tumori Regina Elena, Rome; CR was Senior Investigator and FP acted as Director of the Molecular and Cellular Imaging Unit at the Department of Cell Biology and Neurosciences, Istituto Superiore di Sanità, Rome.

## Supplementary Material

Additional file 1 **Supplemental figure S1. Colocalization of PC-PLC and HER2 on plasma membrane of BT-474 cells**. **(a, b) **CLSM detection of PC-PLC and HER2 in unfixed BT-474 by using rabbit polyclonal α-PC-PLC (green) and α-HER2 W6/100 mAb (red). Colocalization areas are represented in yellow. **(a) **The three-dimensional reconstruction of PC-PLC and HER2 expression on the plasma membrane, and **(b) **the central section. Scale bars, 10 μm. Click here for file

Additional file 2 **Supplemental figure S2. Effect of D609 on HER2 expression in BT-474 cells continuously exposed to trastuzumab**. CLSM analyses on untreated BT-474 fixed cells **(a)** or fixed **(b)** after exposure for the indicated time intervals to trastuzumab (HERC, 10 μg/mL), D609 (50 μg/mL), or their combination (COMB). Nuclei were stained with DAPI (blue). Scale bars, 20 μm. Click here for file

Additional file 3 **Supplemental figure S3. D609-induced retardation of HER2 re-expression on the plasma membrane of SKBr3 cells after short-term receptor engagement with trastuzumab**. CLSM observations on unfixed cells after transient cross-linking with trastuzumab (10 μg/mL, 30 minutes at 4°C), followed by goat α-human FITC-conjugated Ab **(a**, pseudo-color red**)**, then cultured at 37°C for the indicated time periods in complete Ab-free medium, either in the absence **(b** through **f)** or presence of D609, 50 μg/mL **(g** through **m)**. At the end of each time interval, cells were stained again on the plasma membrane with the α-HER2 W6/100 mAb, followed by goat α-mouse Alexa Fluor-594 (pseudo-color green). Nuclei were stained with DAPI (blue). Scale bars, 8 μm. Micrographs represent results of three independent series of experiments performed. Click here for file
