# Supplementary figures and images for: Inhibition of phosphatidylcholine-specific phospholipase C downregulates HER2 overexpression on plasma membrane of breast cancer cells
Source: Breast Cancer Res. 2010 May 12;12(3):R27. doi: 10.1186/bcr2575 (PMC2917016; doi:10.1186/bcr2575)

A

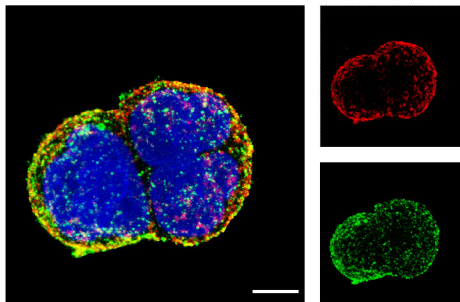

B

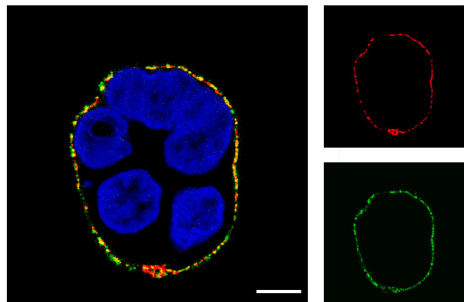

Supplemental FIG 1

Supplement: Additional file 1 — Supplemental figure S1. Colocalization of PC-PLC and HER2 on plasma membrane of BT-474 cells. (a, b) CLSM detection of PC-PLC and HER2 in unfixed BT-474 by using rabbit polyclonal α-PC-PLC (green) and α-HER2 W6/100 mAb (red). Colocalization areas are represented in yellow. (a) The three-dimensional reconstruction of PC-PLC and HER2 expression on the plasma membrane, and (b) the central section. Scale bars, 10 μm. [file bcr2575-S1.PDF]

A

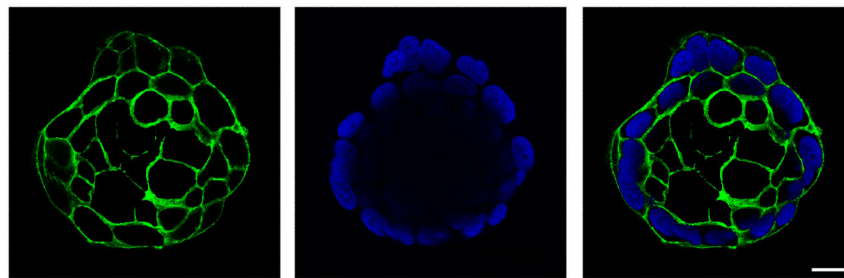

B

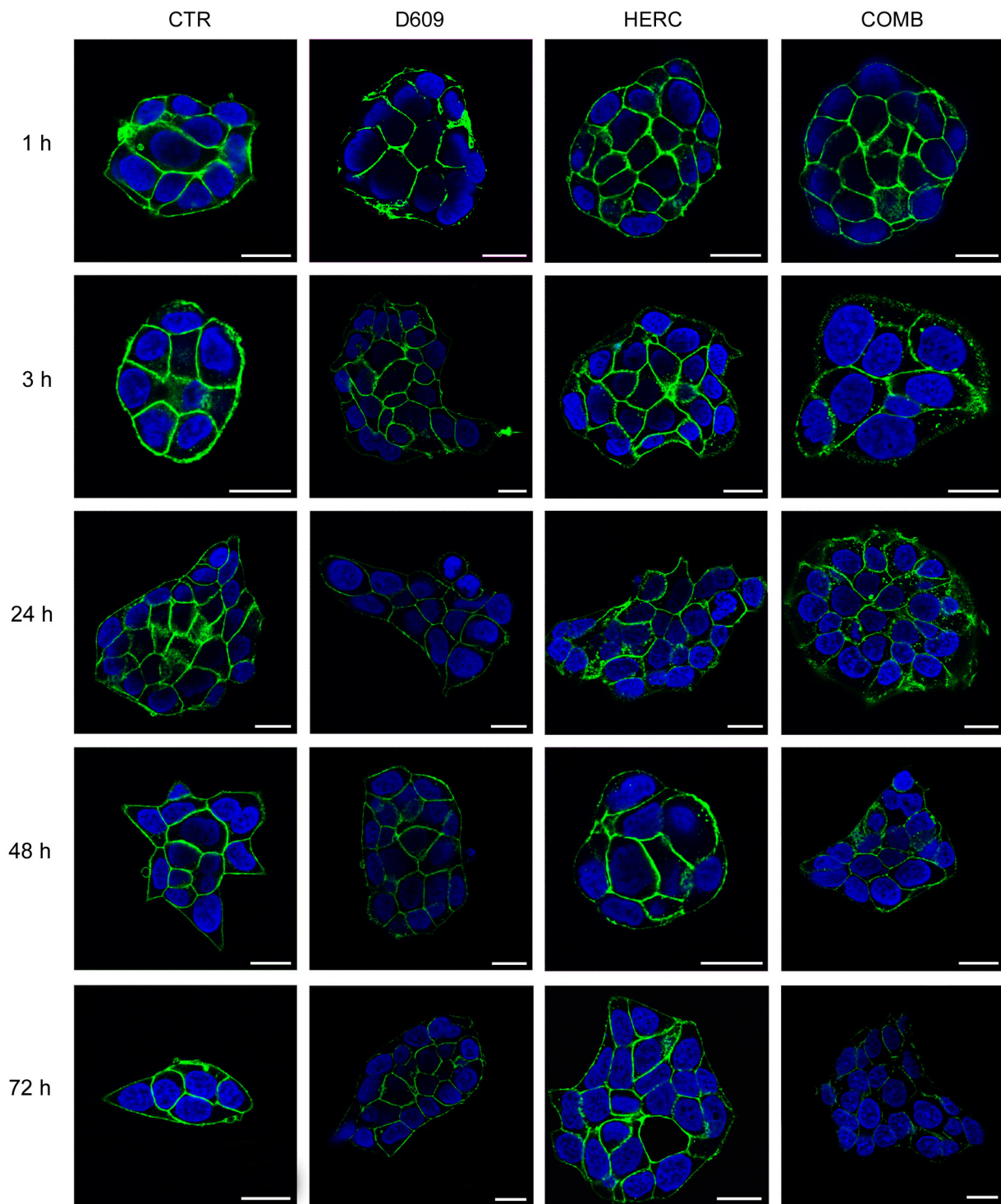

Supplemental FIG. 2

Supplement: Additional file 2 — Supplemental figure S2. Effect of D609 on HER2 expression in BT-474 cells continuously exposed to trastuzumab. CLSM analyses on untreated BT-474 fixed cells (a) or fixed (b) after exposure for the indicated time intervals to trastuzumab (HERC, 10 μg/mL), D609 (50 μg/mL), or their combination (COMB). Nuclei were stained with DAPI (blue). Scale bars, 20 μm. [file bcr2575-S2.PDF]

t0

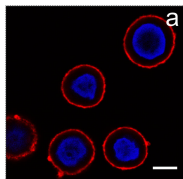

D609

-

+

1h

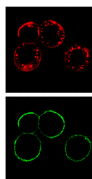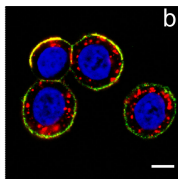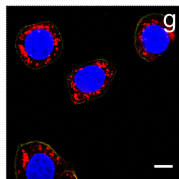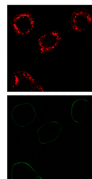

3h

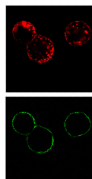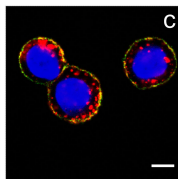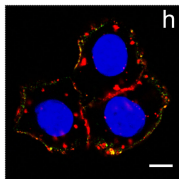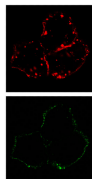

5h

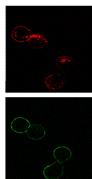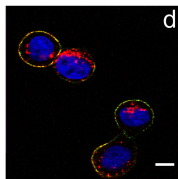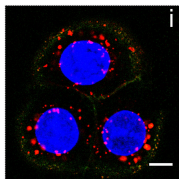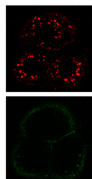

24h

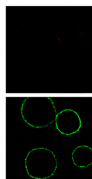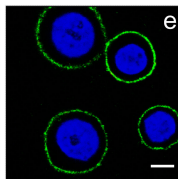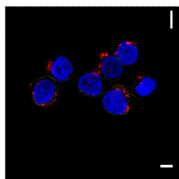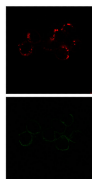

48h

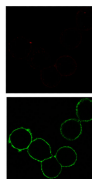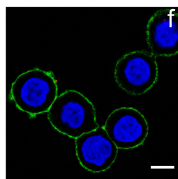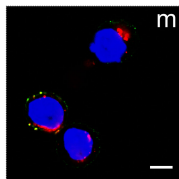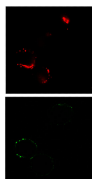

Supplemental FIG 3

Supplement: Additional file 3 — Supplemental figure S3. D609-induced retardation of HER2 re-expression on the plasma membrane of SKBr3 cells after short-term receptor engagement with trastuzumab. CLSM observations on unfixed cells after transient cross-linking with trastuzumab (10 μg/mL, 30 minutes at 4°C), followed by goat α-human FITC-conjugated Ab (a, pseudo-color red), then cultured at 37°C for the indicated time periods in complete Ab-free medium, either in the absence (b through f) or presence of D609, 50 μg/mL (g through m). At the end of each time interval, cells were stained again on the plasma membrane with the α-HER2 W6/100 mAb, followed by goat α-mouse Alexa Fluor-594 (pseudo-color green). Nuclei were stained with DAPI (blue). Scale bars, 8 μm. Micrographs represent results of three independent series of experiments performed. [file bcr2575-S3.PDF]
